# Reliability and validity of six-minute step test in patients with heart failure

**DOI:** 10.1590/1414-431X2020e10514

**Published:** 2021-07-16

**Authors:** R.S. Marinho, S.P. Jürgensen, J.F. Arcuri, C.L. Goulart, P.B. dos Santos, M.G. Roscani, R.G. Mendes, C.R. de Oliveira, F.R. Caruso, A. Borghi-Silva

**Affiliations:** 1 Programa de Pós-Graduação Interunidades de Bioengenharia, Universidade de São Paulo, São Carlos, SP, Brasil; 2 Departamento de Fisioterapia, Universidade Federal de São Carlos, São Carlos, SP, Brasil; 3 Departamento de Medicina, Universidade Federal de São Carlos, São Carlos, SP, Brasil

**Keywords:** Exercise tolerance, Heart failure, Functional capacity, Six-minute step test, Cardiopulmonary exercise testing, Rehabilitation

## Abstract

Exercise intolerance is the hallmark consequence of advanced chronic heart failure (HF). The six-minute step test (6MST) has been considered an option for the six-minute walk test because it is safe, inexpensive, and can be applied in small places. However, its reliability and concurrent validity has still not been investigated in participants with HF with reduced ejection fraction (HFrEF). Clinically stable HFrEF participants were included. Reliability and error measurement were calculated by comparing the first with the second 6MST result. Forty-eight hours after participants underwent the 6MST, they were invited to perform a cardiopulmonary exercise test (CPET) on a cycle ergometer. Concurrent validity was assessed by correlation between number of steps and peak oxygen uptake (V̇O_2_ peak) at CPET. Twenty-seven participants with HFrEF (60±8 years old and left ventricle ejection fraction of 41±6%) undertook a mean of 94±30 steps in the 6MST. Intra-rater reliability was excellent for 6MST (ICC=0.9), with mean error of 4.85 steps and superior and inferior limits of agreement of 30.6 and -20.9 steps, respectively. In addition, strong correlations between number of steps and CPET workload (r=0.76, P<0.01) and peak V̇O_2_ (r=0.71, P<0.01) were observed. From simple linear regression the following predictive equations were obtained with 6MST results: V̇O_2_ peak (mL/min) = 350.22 + (7.333 × number of steps), with R^2^=0.51, and peak workload (W) = 4.044 + (0.772 × number of steps), with R^2^=0.58. The 6MST was a reliable and valid tool to assess functional capacity in HFrEF participants and may moderately predict peak workload and oxygen uptake of a CPET.

## Introduction

Exercise intolerance is a cardinal symptom and the most important clinical characteristic of patients with chronic heart disease (HF), especially in patients with reduced ejection fraction (HFrEF) (1). The assessment of cardiorespiratory adjustments during physical exercise provides useful prognostic information and a basis to correct exercise prescription and propose interventions in this population (1,2). Cardiopulmonary exercise testing (CPET) is considered the gold standard method to establish exercise capacity and primary limitations, i.e., cardiovascular, ventilatory, or peripheral limitations (3-5). On the other hand, CPET is a complex test and requires controlled environment, costly equipment, and trained staff, often being impracticable for a physiotherapy practice (6).

In this context, field tests have been extensively studied and provide an alternate exercise-intolerance assessment, which can be useful in assessing functional capacity, as well as to prescribe physical training (7,8). The six-minute step test (6MST) is a promising alternative for patients with cardiorespiratory diseases (9,10) since it evaluates exercise capacity through the step-climbing activity, an activity required in daily living. In addition, when compared to other common field tests, such as the six-minute walk test and incremental shuttle walk test, the 6MST requires less space and favors monitoring (i.e. electrocardiography). Moreover, by adding movements that require vertical displacement of the body, the 6MST promotes higher oxygen uptake (V̇O_2_) in a shorter period of time and leads to greater cardiovascular stress compared with the six-minute walk test (11,12).

The 6MST has been studied in healthy individuals (11) and in patients with chronic obstructive pulmonary disease (9) and coronary arterial disease without HFrEF (10). However, these authors used submaximal tests for validation criteria. The 6MST was feasible, reliable, and a valid assessment of functional capacity in some populations (9–14). However, reliability and concurrent validity with the gold standard of this test in patients with HFrEF still have not been investigated. Therefore, the primary purpose of the present study was to test the reliability and concurrent validity of the 6MST in HFrEF patients. The secondary purpose was to establish equations to estimate peak V̇O_2_ and maximum workload in this population using the 6MST. The main hypothesis of the study was that the 6MST is a reliable and valid test to evaluate exercise capacity in this population and the number of steps can predict peak V̇O_2_ as well as estimate the workload.

## Material and Methods

Participants with a HFrEF clinical diagnosis on their health records were contacted at the cardiology clinic of the São Carlos Center for Medical Specialties, University Hospital of the Federal University of São Carlos and the Health School Unit of the Federal University of São Carlos. By a telephone call, participants were asked if they i) had an echocardiogram with signs of HfrEF, ii) used beta-blockers, and iii) had a history of infarction, arterial hypertension, or diabetes. When any of these criteria was met, they were invited to participate in the study, starting with a cardiologic consultation and an echocardiogram (Philips HD11, USA) to confirm HFrEF diagnosis and severity. They were excluded if they i) had ejection fraction higher than 51% (women) or 52% (men) (15,16), ii) had been hospitalized in the prior 90 days, and iii) had comorbidities that could impair the 6MST application, such as orthopedic lower limb conditions, stroke, Parkinson's disease, and Alzheimer's disease. Lastly, spirometry (Breeze^®^, Medgraphics, MGC Diagnostics Corporation, USA) was performed to exclude participants with pulmonary diseases (17).

The experimental procedures were divided into two days in the afternoon, with an interval of 48 h between the first and the second visit. On the first visit, participants responded to an interview with personal data, were classified according to the New York Heart Association classification (NYHA) (18), and performed the 6MST twice. On the second day, participants performed the CPET.

### Six-minute step test

The 6MST was performed using a portable 20-cm-high step with a non-slip rubber surface, and the counting of steps was performed by one of the physiotherapists. After 30 min of completing the first test, participants underwent a second test, which was conducted by the same investigator in order to verify intra-rater reliability (19).

Prior to the test, participants were instructed to remain seated at rest for five minutes while the physiotherapist explained the test to the participant (19). Participants were instructed to climb up and down as many times as possible for six minutes regulating their own rhythm (19).

The test was based on the criteria of the American Thoracic Society (ATS 2002) (20) and the physiotherapist who conducted the test was responsible for saying standardized encouragement phrases, informing participants with each remaining minute until completion. Phrases like “you're doing great” and “keep it up” were told, and when the timer was within 15 s of completing the test, participants were told “you shall stop when I ask you to” (19,20).

Ventilatory, cardiovascular, and metabolic measurements were obtained using a gas-analyzer Oxycon Mobile^®^ system (Mijnhardt/Jäger, Germany). Oxygen uptake (V̇O_2_) was calculated at the last 30 s prior to test interruption. Ventilation (V̇E), respiratory rate (RR), carbon dioxide production (V̇CO_2_), and respiratory exchange ratio (RER) were collected breath-by-breath (21). In addition, systolic and diastolic blood pressures (BP) (SBP and DBP, respectively) were verified by the Korotkoff auscultatory method before and at the end of the test. Heart rate (HR) was monitored by means of a heart rate monitor (Polar^®^ S810i, Finland) before, throughout the test, and at recovery. Dyspnea and lower limb fatigue responses were obtained by the Borg visual scale.

Peripheral oxygen saturation (SpO_2_-, Nonin^®^ Plymouth, USA) and 12 lead-echocardiogram (Wincardio^®^, Micromed, Brazil) were continuously monitored during the test. If SpO_2_ dropped below 89% or high-frequency ventricular arrhythmias, ventricular conduction blocks, or signs of ischemia were identified during the test, the test was interrupted. If patients presented dyspnea or limiting lower limb fatigue to perform the test and asked to rest, the test was interrupted and resumed within six minutes. The total number of steps reached at six minutes was computed for data analysis and pauses were recorded.

### Cardiopulmonary exercise testing

Forty-eight hours after both 6MSTs, participants underwent a symptom-limited CPET on a cycle ergometer (Corival Recumbent, Lode BV, Netherlands), in which a continuous incremental ramp protocol was adopted with a workload increase ranging from 5 to 10 watts (W), according to the guidelines on the cardiopulmonary stress test (22). The test was administered by an experienced physician and physiotherapist. The same 6MST variables were evaluated on the CPET, and the same gas analyzer was used. During the test, participants remained seated at rest on the cycle ergometer for five minutes, followed by a one-minute warm-up period without imposed workload (23,24). After this, the test protocol started with an increase in workload every minute, and the participants were instructed to maintain 60 rotations per minute until the onset of symptoms that might suggest discontinuation of the test. Peak values were obtained according to previous studies (22). In general, the test was limited by signs and/or symptoms that indicated the individual's effort, the main reasons being the exhaustion of the peripheral oxygen transport system, and the decrease in the ventilatory and/or metabolic muscle reserve (22).

The test was interrupted when changes in variables related to safety were observed during the CPET, such as SpO_2_ below 89% and SBP equal to or greater than 240 mmHg or DBP equal or greater than 120 mmHg, as well as ventricular arrhythmias such as nonsustained ventricular tachycardia, bigeminated extrasystoles, frequent ventricular extrasystoles, or even ST segment change greater than two millimeters (22,23). All ventilatory and metabolic variables of gas exchange were averaged every 30 s and peak values were defined as the highest value achieved during the test (24).

### Statistical analysis

Twenty-four participants were required based on the sample calculation considering r=0.9 as the expected hypothesis and r<0.7 as the null hypothesis, adopting α=5% and β=20% (25). Shapiro-Wilk test (26) was used to verify the normality of the data, using the SPSS Statistics 20 (IBM, USA) statistical program. Continuous data are reported as means±SD and categorical variables as percentages. Continuous variables were compared with paired Student's *t*-test. Furthermore, P values <0.05 were considered significant.

Intra-rater reliability was verified by comparing the number of steps on the first and second test using a two-way mixed effects intra-class correlation coefficient (ICC), considering a single measurement and the absolute agreement definition. The 6MST would be considered reliable if ICC >0.7. Measurement error was calculated using the minimum detectable difference (MDD), the standard error of measurement, and the Bland-Altman plot (mean error and limits of agreement). The MDD was calculated using the formula (MDD = 1.64 × (√2) × standard error √ (1 - ICC) (11,27,28).

To assess the degree of association between variables, Pearson correlation coefficient was applied for normal data and Spearman correlation coefficient for non-normal data. Correlation strength was classified as 0.3-0.5 = weak, 0.5-0.69 = moderate, and 0.7-0.9 = strong correlation (29). The first 6MST result was used in the concurrent validity analysis, and if its correlation with CPET was r>0.7, it would be considered valid (30). Two simple linear regression models were used to construct prediction equations of both V̇O_2_ and peak workload from CPET, using as independent variable the number of steps on the first 6MST. For the construction of the linear regression equation, we considered the following prerequisites: i) an initial correlation>0.7; ii) a minimum n of 20 individuals for each prediction variable; iii) no auto-correlation between the residuals (or independent residuals), which was verified by a Durbin-Watson test between 1.5 and 2.5; iv) in the ANOVA, the predictor-adjusted model must be different from the model without the predictor (predicted variable with P<0.00); v) the regression model coefficients must have P<0.05; and vi) in the statistical analysis of the residuals, the variation range must be between -3 and +3. In addition, the plots of residuals were analyzed regarding normality of the residuals using a histogram, a PP plot showing the linear relationship of the residual, and a plot evaluating the homoscedasticity of residuals.

Additionally, to verify if V̇O_2_ obtained in a step test was interchangeable with the V̇O_2_ from CPET, agreement between V̇O_2_ obtained in these tests was assessed using a Bland-Altman plot.

## Results

For this study, 182 participants who were being followed-up in the cardiology sector were contacted, based on the medical record. However, 63 did not answer the call, 17 had incorrect telephone contacts in their medical records, 25 subjects refused to participate, and six participants were diagnosed with COPD coexistence, totaling 111 participants excluded from the study.

**Figure 1:**
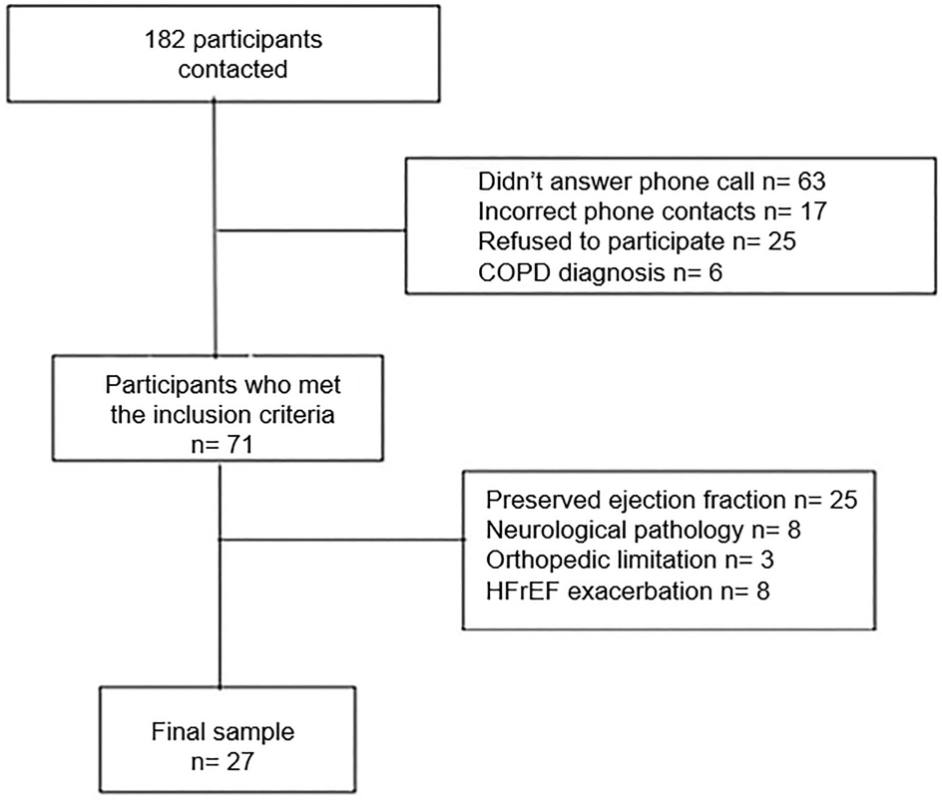
Flowchart of recruited participants. HFrEF: heart failure with reduced ejection fraction; COPD: chronic obstructive pulmonary disease.

Seventy-one participants met the inclusion criteria; however, 25 participants had preserved ejection fraction, eight participants presented disabling neurological disorders, three participants had severe orthopedic limitations, and eight participants presented exacerbation of HFrEF in the previous 90 days and were excluded. Twenty-seven participants completed all evaluations and tests, as shown in the flowchart (Figure 1).

The anthropometric, cardiac, and pulmonary function characteristics of HFrEF participants are described in Table 1. The sample was composed mostly by men, with moderate left ventricular ejection fraction (LVEF) classification, NYHA II classification, and using β-blockers (Table 1).

**Table 1:** Anthropometric characteristics, cardiac function, pulmonary function, and medication of heart failure with reduced ejection fraction (HFrEF) patients.

Variables	HFrEF (n=27)
Age (years)	60±8
Gender n (%)	
Male	16 (59.3)
Female	11 (40.7)
Height, cm	1.64±0.09
BMI (kg/m^2^)	29±5
LVEF (%)	41±6
LVEF, classification n (%)	
Mild	19 (70.4)
Moderate	6 (22.2)
Severe	2 (7.4)
NYHA, I-II-III-IV, n (%)	9 (33) / 11 (41) / 5 (18) / 2 (8)
FEV_1_	2.68±0.70
FEV_1_ % pred	85.9±14
FEV_1_/ FVC	0.79±0
β-blocker, n (%)	27 (100)
ACEi, n (%)	17 (63.0)
Diuretic, n (%)	19 (70.4)
Statin, n (%)	13 (48.1)

BMI: body mass index; LVEF: left ventricular ejection fraction; NYHA: New York Heart Association FEV_1_: forced expiratory volume in one second; FEV_1_/FVC: ratio of forced expiratory volume in one second to forced vital capacity; ACEi: angiotensin-converting enzyme inhibitor. Data are reported as means±SD or number and frequency.

Ventilatory, hemodynamic, and metabolic responses during the first and the second 6MST are reported in Table 2. There was no significant difference between the tests (P>0.05). During the 6MST, 12 volunteers stopped to rest between the second and the third minute. Of these 12 volunteers, seven were men and 5 women who reported fatigue in the lower limbs from three to ten but completed the test before the sixth minute. Only two participants did not complete the test.

**Table 2:** Comparisons between variables at the peak of exercise between the first and second six-minute step test.

	First test	Second test	P
Steps, n	94±30	99±30	0.06
V̇E, L/min	42±17	43±18	0.96
RR, bpm	31±7	32±7	0.50
V̇O_2_, mL/min	1002±395	1033±374	0.69
V̇O_2_, mL·kg^-1^·min^-1^	12±3	12±3	0.44
V̇O_2_, % pred	58±24	61±19	0.43
V̇CO_2_, L/min	1031±387	1053±429	0.57
RER	0.99±1	1.04±1	0.25
SBP, mmHg	154±17	156±27	0.65
DBP, mmHg	93±11	93±11	0.73
HR, bpm	113±19	116±20	0.33
SpO_2_, %	94±2	94±2	0.73
Symptoms (Borg scale)			
Dyspnea (0-10)	3±2	4±2	0.46
Leg fatigue (0-10)	2±2	3±2	0.15

V̇E: ventilation; RR: respiratory rate; V̇O_2_: oxygen uptake; V̇CO_2_: carbon dioxide production; RER: respiratory exchange ratio; SBP: systolic blood pressure; DBP: diastolic blood pressure; HR: heart rate; SpO_2_: oxygen saturation. Data are reported as means±SD (Student's *t*-test).

**Figure 2:**
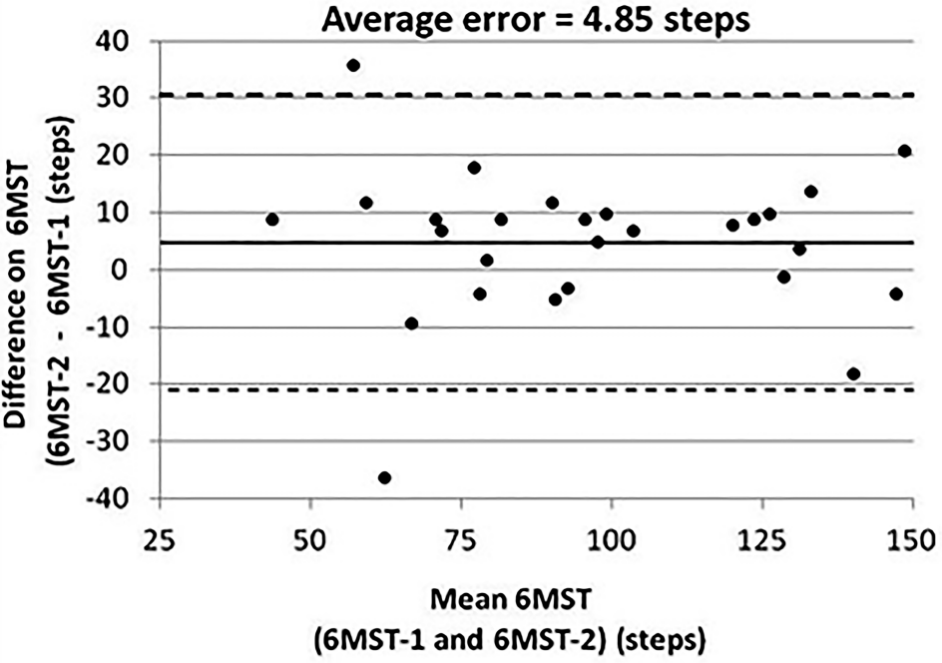
Bland-Altman plot for error measurement analysis between the first and second six-minute step test (6MST) considering as variable the number of steps.

Reliability between first and second 6MST results was excellent, with an ICC=0.9 (95%=0.78-0.95). On the error measurement analysis, the Bland Altman plot (Figure 2) presented a mean error of 4.85 steps (95% LA=30.6 to -20.9). The standard error of measurement was 9.20 steps and MDD was 21 steps.

Variables at the peak of CPET and at the end of the 6MST effort are described in Table 3. As expected, RER was significantly lower as measured with the 6MST compared with the CPET (P<0.02). In addition, lower SBP and DBP were observed during the 6MST when compared with the CPET (P<0.01).

**Table 3:** Ventilatory, cardiovascular, metabolic, and perceptual responses at peak cardiopulmonary exercise test (CPET) and in the six-minute step test (6MST) in heart failure with reduced ejection fraction (HFrEF) patients.

Variables	6MST	CPET	P
Steps, n	94±30	-	-
Workload, watts	-	77±31	-
V̇E, L/min	42±17	46±14	0.57
RR, bpm	31±7	33±6	0.25
V̇O_2_, mL/min	1025±385	1034±311	0.89
V̇O_2_, mL·kg^-1^·min^-1^	12±3	12±3	0.79
V̇O_2_, % pred	58±24	60±21	0.10
V̇CO_2_, L/min	1031±387	1136±377	0.14
RER	0.99±1	1.05±1	0.02
SBP, mmHg	154±17	201±25	<0.01
DBP, mmHg	93±11	112±14	<0.01
HR, bpm	113±19	116±17	0.52
SpO_2_, %	94±2	95±2	0.73
Symptoms (Borg scale)			
Dyspnea (0-10)	3±2	4±2	0.74
Leg Fatigue (0-10)	2±2	3±2	0.16

V̇E: ventilation; RR: respiratory rate; V̇O_2_: oxygen uptake; V̇CO_2_: carbon dioxide production; RER: respiratory exchange ratio; SBP: systolic blood pressure; DBP: diastolic blood pressure; SpO_2_: oxygen saturation. Data are reported as mean and standard deviation (Student's *t*-test).

A male volunteer developed angina during CPET exercise, and the test was stopped. Also, two male patients had SBP higher than to 240 mmHg and the test was interrupted by the team.

**Figure 3:**
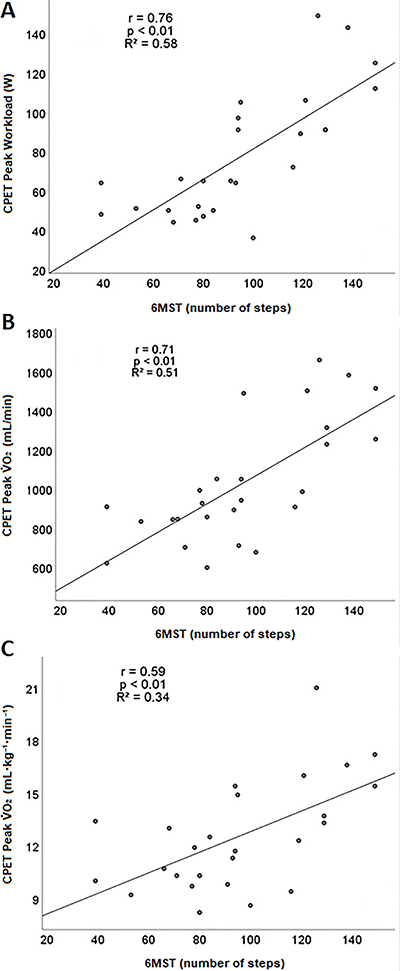
Correlations between (**A**) peak workload on cardiopulmonary exercise test (CPET) and six-minute step test (6MST) number of steps, (**B**) CPET peak V̇O_2_ (mL/min) and 6MST number of steps, and (**C**) CPET peak V̇O_2_ (mL·kg^-1^·min^-1^) and 6MST number of steps.

There were strong correlations between steps obtained in the 6MST and workload (W) in the CPET (r=0.76, P<0.001, R^2^=0.58) (Figure 3A) and between absolute V̇O_2_ (mL/min) at the peak of CPET with step numbers (r=0.71, P<0.001, R^2^=0.51) (Figure 3B). In addition, moderate correlation was found between V̇O_2_ (mL·kg^-1^·min^-1^) and steps obtained in the 6MST (r=0.59, P<0.01, r^2^=0.34) (Figure 3C).

The number of steps obtained in the 6MST predicted peak V̇O_2_ (mL/min) and maximal workload in the CPET (Table 4).

**Table 4:** Predictive model of peak oxygen uptake and workload in the cardiopulmonary exercise test (CPET).

Variables	Coefficient	Standard error	P
Oxygen Uptake			
Constant	350.22	139.75	0.02
Steps	7.333	1.39	<0.01
Workload			
Constant	4.044	13.51	0.77
Steps	0.772	0.13	<0.01

R^2^ adjusted=0.51; (P<0.002). Prediction equation for V̇O_2_ in CPET (mL/min) = 350.22 + (7.333 × steps). R^2^ adjusted=0.58; (P<0.001). Prediction equation for workload in CPET (W) = 4.044 + (0.772 × steps).

**Figure 4:**
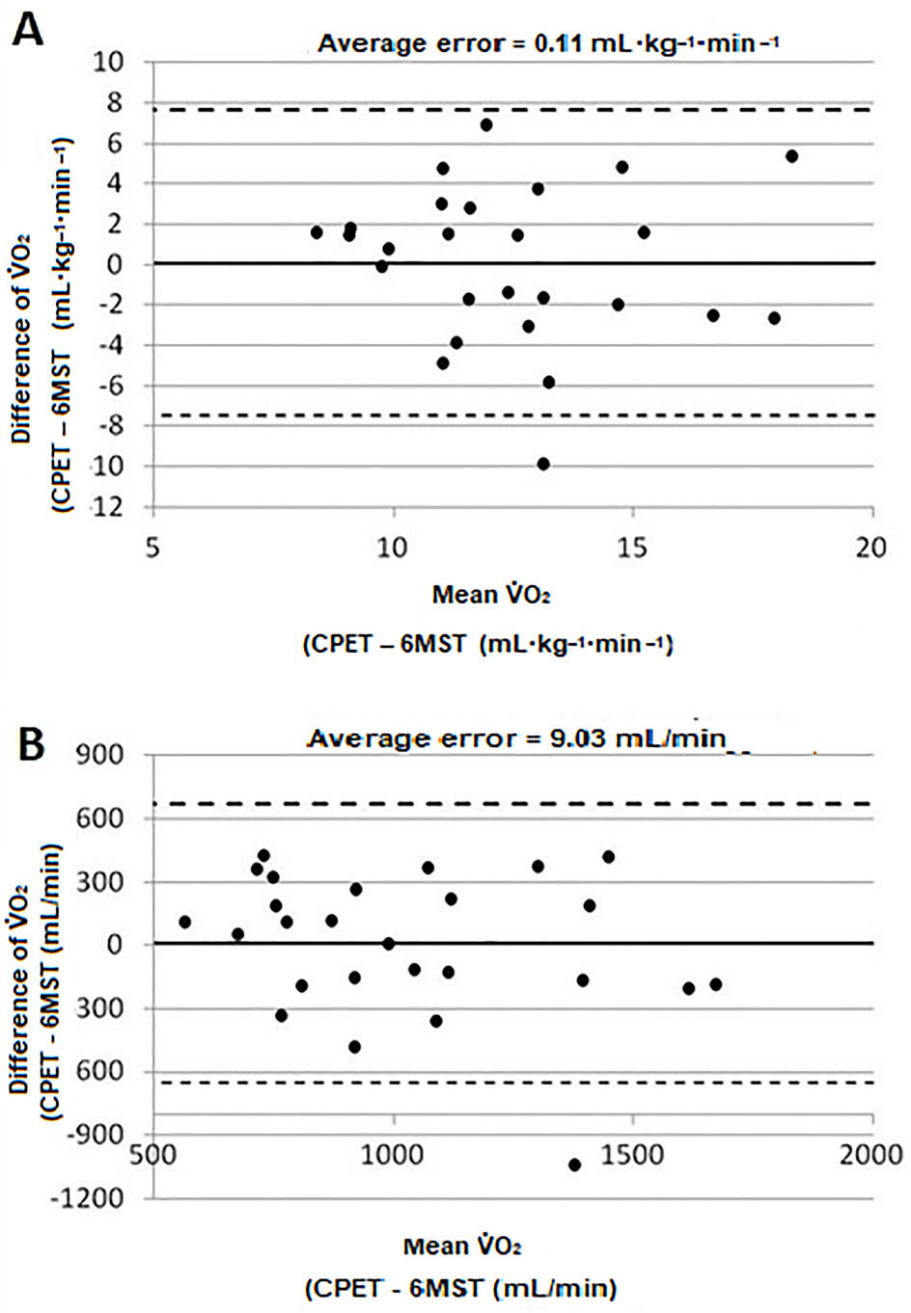
Bland-Altman plot for agreement analysis between six-minute step test (6MST) and cardiopulmonary exercise test (CPET) comparing the variables (**A**) V̇O_2_ (mL·kg^-1^·min^-1^) and (**B**) V̇O_2_ (mL/min).

The plot to assess agreement (Figure 4A) shows a low average error (peak V̇O_2_=0.11 mL·kg^-1^·min^-1^) when comparing peak V̇O_2_ in the CPET and in the 6MST. However, a large limit of agreement was also observed (inferior limit=-7.47 mL·kg^-1^·min^-1^ and superior limit 7.69 mL·kg^-1^·min^-1^) (Figure 4A). A similar result was found when V̇O_2_ (mL/min) was used in the analysis (Figure 4B).

## Discussion

Several aspects make this study a novel investigation. Specifically, this is the first study, to our knowledge, to test the reliability and validity of the 6MST in the HFrEF population. The main findings in this study were: 1) 6MST presented excellent reliability when conducted by the same investigator; 2) the number of steps on 6MST were strongly associated to maximal workload and peak V̇O_2_ (mL/min) on CPET; and 3) the number of steps on 6MST may moderately predict peak V̇O_2_ (51%) and the peak workload (58%), thus findings must be interpreted with care. In this context, self-cadenced 6MST provided estimates of functional capacity in HFrEF participants.

### Reliability of 6MST in HFrEF participants

Considering the reliability analysis between the first and second 6MST, ICC values were above 0.7. Additionally, variables were not different on first and second tests (Table 2), demonstrating excellent reliability of participants with HFrEF. Thus, the performance obtained in the first and second tests were similar, as well as ventilatory, cardiovascular, and metabolic demand. In this sense, it is not necessary to apply two tests to these participants, diminishing the time spent, which favors its applicability.

Our results corroborated those of Arcuri et al. (11) who also analyzed the reliability of the 6MST in healthy individuals and concluded that the non-need of a familiarization test was an advantage of the 6MST over the 6MWT. Dal Corso et al. (31) studied the 6MST’s reliability in interstitial lung disease patients and concluded that there was no statistical difference between the test and retest, supporting the above finding. Recently, Travensolo et al. (10) examined the reliability in coronary artery disease individuals and did not find any difference between the first and second test.

In our study, the MDD was 21 steps, which was similar to the study of Arcuri et al. (11) (MDD=27 steps). These results highlight that healthy individuals and those with chronic diseases require a minimum number of steps to identify clinical improvement. Therefore, the 6MST is a viable and fast alternative to access functional capacity. The test is a reliable tool to measure exercise capacity in various health care settings, since it requires little space for its performance and allows better monitoring and security during its application compared to other field tests.

### Validity of the 6MST in patients with HFrEF

The number of steps on the 6MST presented a strong correlation with CPET peak V̇O_2_ (mL/min), thus, this field test presented concurrent validity to assess functional capacity in the HFrEF population. Moreover, it was possible to predict V̇O_2_ using the number of steps as the independent variable, which would help patient prognosis (32,33). The number of steps and CPET peak V̇O_2_ as a ratio of body weight (mL·kg^-1^·min^-1^) presented a moderate correlation (r=0.59, Figure 3C), and the expected drop in correlation coefficient may be explained by the influence of body mass on the number of steps in the 6MST (11).

Impaired cardiorespiratory fitness measured by V̇O_2_ peak is an important manifestation of HFrEF (22). In addition, V̇O_2_ peak can predict morbimortality in HFrEF participants. However, CPET is costly and requires specialized staff. For this reason, field tests may be a simpler and more viable alternative to assess functional capacity as it requires fewer evaluators and is a low-cost test.

Although 6MST was valid to assess functional capacity, V̇O_2_ at the end of this test did not agree with CPET peak V̇O_2_ (Figure 4), which indicates V̇O_2_ obtained with the 6MST may not be used as a substitute of V̇O_2_ obtained with the CPET. This finding contrasts with the study of Dal Corso et al. (31), in which the error was smaller. Hence, in a situation when a CPET is not available to assess functional capacity of patients with HFrEF, the use of the estimated values based in the number of steps should be preferred.

Another important finding of our study was that the CPET workload was strongly associated with the 6MST number of steps, allowing the prescription of exercises on a cycle-ergometer for this population based on number of steps achieved (Table 4) (32–34).

### Physiological responses with the 6MST and CPET

Ventilatory demand and symptoms were similar between tests. However, peak blood pressure and RER were higher during the CPET than the 6MST (22). Therefore, the CPET workload induces cardiovascular responses in this population that might present more risks during an exercise assessment (32–34).

We obtained lower values of SBP and DBP at the end of the 6MST test compared to CPET, which indicated less cardiovascular stress (Table 3). Thus, despite the 6MST being an exhaustive test, it does not lead to maximum cardiovascular stress. Therefore, it should be characterized as a submaximal test in HFrEF participants, being a safer test to be conducted in clinical practice. Nevertheless, the CPET still provides wider information regarding why functional capacity may be impaired in patients with HFrEF. The CPET better assesses hypertensive responses to exercise, which is important to help clinical decision making, as high SBP responses during exercise have been linked to worse cardiovascular outcomes in patients with HF, such as occurrence of angina and arrhythmogenic events (35).

A significant proportion of patients interrupted the test due to lower limb fatigue. These results were observed previously in other chronic disease populations (32–34).

### Limitations

This study has some limitations. Firstly, our findings are limited to the population with HFrEF and cannot be extrapolated to HF with preserved LVEF. Secondly, reliability cannot be extrapolated to inter-rater comparisons, as only one physiotherapist performed the step count. Third, no previous study assessed if different intervals between tests would improve reliability. Fourth, although the present study met all the prerequisites to assume that the number of steps can predict V̇O_2_ (51%) and the peak workload (58%) of the maximum exercise test, other factors such as weight, gender, and age can also affect V̇O_2_ variation. Care must be taken when predicting peak V̇O_2_ from the 6MST because CPET is the gold standard test. Future studies with a larger sample can test new equations with higher prediction capacity considering these variables. Finally, although the 6MST is a valid tool to assess functional capacity, the CPET is the gold standard for heart transplantation indication, prognosis, and rehabilitation in this population. Thus, we encourage future research to assess the validity and reliability of the 6MST for assessing functional capacity in other populations with HF.

### Conclusion

The 6MST was a reliable and valid tool to assess functional capacity in HFrEF participants and may moderately predict peak workload and oxygen uptake of a CPET.
